# Short-Term Effects of a Multidimensional Stress Prevention Program on Quality of Life, Well-Being and Psychological Resources. A Randomized Controlled Trial

**DOI:** 10.3389/fpsyt.2019.00088

**Published:** 2019-03-12

**Authors:** Romina Evelyn Recabarren, Claudie Gaillard, Matthias Guillod, Chantal Martin-Soelch

**Affiliations:** Division of Clinical and Health Psychology, IReach Lab, Department of Psychology, University of Fribourg, Fribourg, Switzerland

**Keywords:** stress, stress management, intervention program, psychological distress, anxiety, psychological resources, quality of life, university students

## Abstract

It is well-documented that university students have an increased risk in developing psychological problems because they face multiple stressors. Cognitive, behavioral, and mindfulness-based stress prevention programs were shown to reduce symptoms of anxiety, depression, and perceived stress in university students. However, little is known of their effect on resource activation. Additionally, most validated interventions are unidimensional, i.e., including one stress-coping approach. In this study, we investigated the short-term effects of a multidimensional stress prevention program on students' quality of life, psychological symptoms and resources, and resilience factors against stress. Using an experimental design, 64 healthy undergraduate students (56 women), between 18 and 34 years old (M = 21.34, *SD* = 2.53), from the University of Fribourg, Switzerland, were randomly allocated either to the intervention or the wait-list control group. The intervention group participated in a multidimensional stress prevention program, integrating mindfulness-based activities, cognitive and behavioral strategies, social skills, and emotional regulation exercises. The program consisted of eight 2-h weekly sessions. Before and after the intervention, participants completed self-reported questionnaires evaluating quality of life; psychological symptoms such as depression, anxiety, social anxiety, and interpersonal problems; as well as psychological resources like self-efficacy, sense of coherence, self-compassion, and social support, presented online. A standardized clinical interview was performed at pre- and post-measurement times. To analyze the sort-term effects of the program, we used mixed, two-factorial ANOVAs (per-protocol analyses). In accordance with our hypotheses, our results showed significant reduction of psychological symptoms, including anxiety, interpersonal problems, and symptoms of pain; a significant increase in quality of life, sense of coherence, and self-compassion in students who participated in the intervention program compared to the control group, (all *p* < 0.05). No significant results were found for symptoms of depression, social anxiety, self-efficacy, and social support. These preliminary findings indicate specific short-term effects of our multidimensional stress prevention program on psychological symptoms and on quality of life as well as promising effects on psychological resources and factors associated with resilience against stress. Future studies should investigate the long-term effects of the intervention as well as the effects in clinical samples.

## Introduction

University studies are a motivating step in life, yet at the same time students have to face new challenges and circumstances (1). The transition to university life requires them to adapt to a new academic environment with unfamiliar assessment rules and a heavier workload (2, 3). Additionally some students have jobs to make their financial needs meet or move away from friends and families in order to study in other cities (1, 2). Students therefore have more freedom and autonomy, but also other responsibilities and sometimes fewer resources (e.g., social support) (1, 2). According to recent studies, university students reported increased psychological distress in different countries worldwide. In particular, higher prevalence of psychological distress was reported in medical students in Germany (3–5), the US (6), and Egypt (7). According to the results of a survey of the American College Association (8), around half of the Canadian students reported depressive and anxiety symptoms during the last year. Studies in the UK, Spain, Jordan, and India indicated that nurses and dental students showed high levels of distress (9–12). Longitudinal studies in the US revealed that the first year of study is associated with particularly elevated psychological distress in college students (1, 13). Finally, university students were shown to experience higher psychological stress levels than their peers in the general population. For instance, in Australia, university students showed higher levels of distress than non-students (14), and than the general population (15). The most frequent stress factors cited by university students are related to their studies and academic demands (e.g., exams, assessments, assignments, practicum) (2, 16), personal and social expectation (17), living conditions, and financial situation (18, 19).

Psychological distress in university students is associated with increased mental health disorders [31,4% of 12-month prevalence of any mental disorder in first year students from eight countries (20)], such as depression (21–25), and anxiety (22, 25). Burnout (26), suicidal ideation (22, 27), suicide attempts, and self-injurious behavior (28) were also reported in this population. Somatic complaints (29, 30), and physical illnesses, such as skin symptoms (31) and functional gastrointestinal disorders (32) were also manifested by university students. Substance abuse, such as high consumption of alcohol (33, 34), tobacco (smoking), and cannabis (2) were also related with high levels of distress. Higher levels of psychological distress are negatively correlated with student's academic performance (35, 36), such as slipping grades. Poor quality of life (37) and well-being were also reported by university students (38). Sleep disturbances (30, 39–42), unhealthy lifestyle behaviors (e.g., poor nutrition, physical inactivity) (43, 44), fewer leisure activities and less social support, especially during the preparation and examination period (45) were described by university students. Students also reported using more avoidance (46) and withdrawal coping strategies (2), and less adaptive coping strategies, like social support (2, 47), cognitive reappraisal, and planning (48).

Personal and psychosocial resources were found to have a protective role against stress in university students (3). High levels of self-efficacy in university students were associated with less burnout, emotional exhaustion (49), perceived stress (3), and also with positive effects on grades (50), a more proactive attitude, and a better use of available support (49). In a study with French college students, self-efficacy was one of the most important predictors of stress (25).

A strong sense of coherence is related to good stress management and has an impact on the quality of life in different populations (51), including university students (52). A high sense of coherence was negatively associated with perceived stress (53, 54) and positively related with better social support and performance (53), and the use of active coping (55) among university students. Self-compassion, being kind and understanding toward oneself in negative circumstances, predicted greater well-being (56) and correlated significantly with positive mental health outcomes, such as less depression and anxiety and greater life satisfaction in undergraduate students (57). Perceived social support has also been associated with fewer stress symptoms, anxiety, and depression and with higher levels of resilience among university students from different countries (Germany, Russia, and China) (21).

Although university students report increased levels of psychological distress, only a minority of them seek help (15, 58, 59). In the past few years however, diverse stress reduction interventions for university students have been proposed. In a review and meta-analysis, Regehr et al. (60) showed that cognitive, behavioral, and mindfulness-based interventions aiming at reducing stress in university students were associated with decreased symptoms of anxiety, depression, and cortisol levels. Twenty-four randomized controlled studies, including 1,431 students (24% male), were considered for the analysis. Taken together, the analyzed intervention had a significant impact in the reduction of symptoms of anxiety in the experimental groups compared to the control groups. Furthermore, both cognitive-behavioral (CBT) and mindfulness-based interventions showed an improvement in anxiety levels.

Mindfulness is characterized by paying attention in the present moment, non- judgmentally, with self-awareness, and is related to the reduction of stress perception and stress-related symptoms (61). Mindfulness-based interventions were shown to have an impact on stress during the examination period (62, 63), as well as on perceived stress, mental distress, well-being and self-efficacy among medical students (64, 65), and self-compassion in undergraduates students (66). In some studies, however, mindfulness-based interventions had significant beneficial effects on psychological morbidity, but not on distress or coping (67). Other studies indicated that there were no significant differences between the effects of a mindfulness-based group compared to a physical activity program in reducing anxiety, depression and stress (68). With regard to the CBT interventions aiming at reducing stress, they are generally focused on awareness of automatic thoughts; on understanding of the relationship between thoughts and emotions, on cognitive restructuring, on problem-solving, on self-instructions, and on relaxation techniques. For these interventions, a significant impact on anxiety (69–72), on anger and neuroticism (69), on somatic symptoms and cortisol levels (70, 73), on hardiness, and on general self-efficacy (71) was reported in undergraduates students. A significant effect was found also on hope, but not on the amount of self-reported positive or negative affect (72). Finally, a strength-based CBT intervention showed significant improvements on distress, on protective factors, and on quality of life in first year psychology students (74). This specific intervention was focused on improving resilience skills, by activating personal strengths and talents. Other stress prevention programs included social cognitive methods, also including exercises on communication skills, and were shown to reduce psychological distress among university students (75). Relaxation-based interventions, focusing on autogenic training and progressive muscle relaxation, also demonstrated significant effects on cognitive and emotional burnout stress, on trait anxiety, and on mental health in university students (76–78). Finally, an intervention focusing of resources, the Resilience and Coping Intervention, showed significant beneficial effects on optimism, hope, stress, and on depression in undergraduate students (79). However, to our knowledge, only one stress reduction program for college students integrated a multidimensional program including psychoeducation, cognitive reconstructing, emotional control exercises, and communication skills (80). This intervention showed a significant decrease in psychological distress, but no effects on coping strategies or on cortisol levels. In conclusion, to date, no stress prevention intervention for students integrates all dimensions of stress, i.e., behavioral, cognitive, emotional, and social at the same time; focusing not only on stress management mechanisms but also on improving stress protection resources.

In this study, we aimed to evaluate the short-term effects of a multidimensional stress prevention program integrating mindfulness-based activities, cognitive, and behavioral strategies, social skills and assertiveness activities, and emotional regulation exercises on indicators of quality of life, psychological symptoms, well-being, and psychological resources in university students. We compared the outcome variables in an intervention group and a wait-list control group before and shortly after the end of the program (2 months later). We expected significant decreases in psychological symptoms, including depression, anxiety, pain, social phobia, and anxiety symptoms, a significant increase of quality of life and of psychological resources, including self-efficacy, sense of coherence, self-compassion, and social support in the intervention group compared to the control group.

## Materials and Methods

### Participants

Participants were recruited at the University of Fribourg, Switzerland. Data collection was carried out between March 2015 and May 2017. The recruitment was made by e-mail, which were sent to all students of the university (*N* = 10,000), flyers, presentation of the study in diverse classes, webpages of the student's groups, and by word of mouth. The majority of students interested in participating in this study contacted us by e-mail. We answered all the questions and sent the students a document with all the detailed information about the study. Interested students were contacted by phone to explain the study in more detail. A first interview was scheduled as soon as the students accepted to participate in the study, during which exclusion and inclusion criteria were tested.

**Figure 1:**
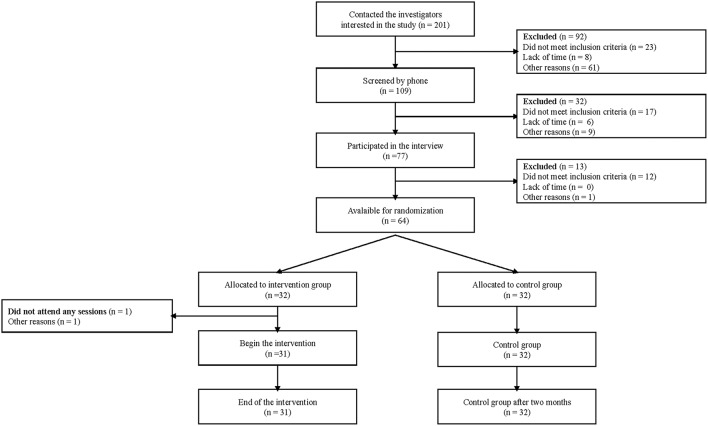
Participant flow chart from recruitment until post-treatment measures.

Initially, 201 students (around 2% of all university students) contacted us to participate in the study; and, to be eligible to the study, participants had to be a university student and understand French. Criteria for exclusion included the presence of an existing mental disorder or endocrinal disease, or brain injury or neurological disorder, and the use of psychotropic drugs. Moreover, participants were excluded if they underwent any type of therapy or coaching at the moment of the study (11 students). After the interview (14 students) withdrew from the study due to lack of time. Figure 1 shows the participants flow diagram of the study.

The final sample was composed of sixty-four university students aged between 18 and 34 years (*M* = 21.34, *SD* = 2.53); 87.5% were women and 68.8% were native French speakers or spoke French fluently (see Table 1). The majority of the participants studied psychology (79.7%) and the other fields of studies were pedagogy (4.7%), law, economy, history, social work, Slavic studies, informatic, French, neurobiology, nursing care, and business communications. Only one person was married, the majority single, 68% were alone and 25 % living with a partner. The majority of participants (49%) were in a medium socioeconomic position, according to the IPSE Index (81). Four cohorts of 16 participants were recruited in each semester. No significant differences were found in the sociodemographic variables between the participants of the wait-list control group and of the intervention groups (all *p* >0.05) [age: *t*_(62)_ = −0.393, *p* = 0.696; sex: *X*^2^(1) = 0.571, *p* = 0.450; socioeconomic position: Cramer's *V* = 0.135, *p* = 0.769; studies (psychology and other): *X*^2^(1) = 0.097, *p* = 0.756]. Eight students reported past psychopathological problems. None of the participants was receiving a treatment (neither drug or psychological) at the time of the study.

**Table 1:** Characteristics of participants (*n* = 64).

	**Intervention group**	**Wait-list control group**
	***n* = 32**	***n* = 32**
Age Mean (*SD*)	21.22 (2.27)	21.47 (2.8)
Gender (Females)	29 (90.6 %)	27 (84.4%)
Mother tongue (French)	21 (65.6%)	23 (71.9 %)
Marital status (single)	25 (78.1%)	19 (59.4%)
Single with partner	6 (18%)	10 (31%)
Living with partner	1 (3.1%)	2 (6.3%)
IPSE score [36–80] (middle class)	23 (76.67)	26 (81.25%)
Studies (Psychology)	26 (81.3 %)	25 (78.1 %)
Bachelor students (First year)	11 (34.4%)	13 (40.6%)
M.I.N.I. DIAGNOSTIC (LIFETIME)		
Past Depression	1 (3.1%)	4 (12%)
Panic Disorder	1 (3.1%)	1 (3.1%)
PTSD		1 (3.1%)

*SD, Standard Deviation. Age in years. IPSE, Indice de position socioéconomique; BA, Bachelor; M.I.N.I, Mini-International Neuropsychiatric Interview; PTSD, Post-traumatic stress disorder*.

### Procedure

Using a randomized-controlled design, this study compared an intervention group who participated in a multidimensional stress intervention program with a wait-list control group. The control group underwent the same measurements at the same measurement times as the intervention group but did not follow the program, nor did they receive another treatment. The outcome variables were assessed before (T1) and after (T2) participation in the program in both groups. The study's protocol was accepted by the Ethics Committee of the Cantons of Vaud and Fribourg (Protocol 261/14). All participants received detailed information about the purpose and the study's process and signed a written informed consent. Confidentiality was guaranteed and participants could withdraw from the study at any time. This research followed the ethical principles of the Declaration of Helsinki (82) and local regulatory law. For this protocol, we also followed the guidelines SPIRIT (83). This study was registered in the research register of the University of Fribourg FUTURA (Project number 6239; http://admin.unifr.ch/futura/content/projects/6239) as well as in the Clinicaltrial Register (https://clinicaltrials.gov. NCT03861013).

After having signed the written informed consent, students participated in a structured interview, the Mini-International Neuropsychiatric Interview [M.I.N.I., (84). French version, (85)], conducted by a psychologist or by trained masters students in psychology, which took between 30 and 60 min. When consent was given, the interviews were filmed. The interviewers were blinded to the group allocation. Following the interview, self-reported online questionnaires were sent and the participants had to complete them in the following days. The participants received a link to access to online questionnaires in an e-mail, and they received their participant's code in a separate text message. When completing the questionnaires using the survey program (LimeSurvey GmbH, Hamburg, Germany. http://www.limesurvey.org), the participants had to enter their codes. The online questionnaires took the participants ~1 h, with the possibility to take breaks whenever needed. Pre-measurements were completed at no more than 2 weeks before the beginning of the study (T1). After that, participants were randomly distributed in the intervention or the wait-list control group. The randomization was done using a free available software, i.e., www.randomization.com and was archived in an electronic document saved separately. After randomization had been done and due to the design of the study, investigators and participants were not blinded about group allocation. The duration of the program was 2 months. Post-measures were taken after a maximum of 2 weeks after the end of the intervention (T2) and included a structured interview and the self-reported online questionnaires. In all cohorts, T2 measurements were always performed at the end of the semester. For their participation, the students received money (CHF 100) or experimental hour compensation (for psychology students). All the data collected were deidentified with a code and confidentiality guaranteed. Participants did not have a dependent relationship with the research team, as the researchers were not involved in teaching of bachelor students. Once the study was completed, participants in the wait-list control group were given the possibility to participate in the program if they wished so, but finally none of them participated, because of lack of time.

## Intervention

A multidimensional stress prevention program integrating mindfulness-based activities, cognitive and behavioral strategies, social skills exercises, and emotional regulation was proposed to the students. This intervention was composed of eight modules and integrated validated techniques from different approaches (Freiburger Training gegen Leistungsstress (86) including cognitive behavioral techniques; RFSM-e-MOTION (RFSM, Réseau Fribourgeois de Santé Mental, i.e., Fribourg Mental Health Network). The RFSM-e-MOTION intervention is a validated online program for relatives of individuals with mental disorders that focuses on the emotional aspects of the family members' experiences and their relationship with the suffering person [(87)., see http://rfsm-e-motion.ch]. This program is based on Dialectical behavioral therapy (88).

The intervention consisted of eight 2-h weekly group sessions. The groups were composed of a maximum of eight students and were led by two trained clinical psychologists. Homework between sessions was also proposed. Participants received the activities printed or on a CD. During the first session, participants presented themselves, the rules of the group functioning were discussed and a confidentiality document was signed. Then, personal experience of stressful situations, triggered emotions, coping strategies, and their efficacy were discussed. The participants' experience with stress was the basis to introduce theoretical information about the topic. Each session followed the same structure. From the second to the last, we always started with a brief breathing exercise. A summary of the former meeting and the objectives of the new session were presented. Afterwards, a review of the homework was done before starting with the new content. At the end of each session, homework was proposed and participants answered questionnaires about group cohesion and the therapeutic alliance. Sessions 2 to 4 addresses behavioral and cognitive techniques (e.g., breathing exercises; planning and cognitive restructuring) and also mindfulness-based exercises (e.g., awareness of breath meditation; exercises for living at the present moment). Sessions 5 and 6 addressed the topic of emotions and emotion regulation. Sessions 7 and 8 integrated assertiveness training and social skills components (e.g., validating communication; interpersonal conflict resolution) (see Table 2 for a session overview).

**Table 2:** Content of the program.

**Session**	**Content**
1	Organizational matters and program overview. Stress, triggers, and coping strategies
2	Cognitive and body techniques, mindfulness-based exercises
3	Cognitive and body techniques
4	Cognitive techniques
5	Emotion and emotion regulation
6	Emotion regulation
7	Social skills and assertiveness
8	Social skills and assertiveness. Evaluation of the program and personal goals

The objective of this program is not only to experiment several techniques to prevent and to cope with stress, but also to increase resources of being more resilient against stress that the participants could use as psychological tools in their everyday life. The intervention was intended to be as experiential as possible. Participants sometimes worked alone, in pairs, in subgroups or in plenum. They performed written exercises, discussions, and role playing in personal or fictive situations. Different types of material and triggers were also used, such as videos, audio, and visual supports. At the end of each exercise, a plenary discussion and a short theoretical link was made. The program was manualized, and each session was protocoled by a masters-level student to ensure compliance with the program. Throughout the entire program, external psychotherapists were available for supervision when needed.

### Measures

All students participated in a structure diagnostic interview the Mini-International Neuropsychiatric Interview [M.I.N.I., (84). French version, (85)] in order to exclude participants with *psychopathological disorders*. This short-structured interview assesses DSM-IV (89) and ICD-10 (90) psychiatric disorders. At T1 we used the lifetime version, and at T2, the current one.

Psychological symptoms and quality of life were measured with the following self-reported instruments that were presented online using LimeSurvey® (LimeSurvey GmbH, Hamburg, Germany. URL http://www.limesurvey.org)

*Sociodemographic information*. At first, participants were asked to report age, sex, marital status, nationality, languages (mother tongue and the language used at home), studies, and grade level. They also answered the *Indice de position socioéconomique* (81) which provides an index on the socio-economic position of the participant in relation to the Swiss population.

A screening for *mental health problems* was done with the French version of the Symptom Checklist [SCL-27-plus, (91)]. Composed of 27 items rated on a 5-point Likert-like scale, this checklist evaluates five dimensions: depressive, vegetative, agoraphobic, and social phobia and pain symptoms, and a global severity index. A lifetime assessment for depressive symptoms and a screening question for suicidality are also included. Cut-offs: social phobia = 1.86; vegetative = 1.54; pain = 1.77; agoraphobic = 0.93; actual depression = 1.28). Cronbach's alpha coefficient in this study were from 0.52 to 0.86.

*Depression* was measured with the Beck Depression Inventory - II [BDI-II, (92)]. Composed of 21 items, this inventory assesses the intensity and severity of depressive symptoms over the past 2 weeks. Items are rated in majority on 4-point Likert-like scale, from zero to three. Higher scores indicate severe depressive symptoms. Score thresholds from 12 to 19: mild = depression, 20 to 27 = moderate depression, and >27 = severe depression. In this study the Cronbach's alpha coefficient was 0.84.

The State-Trait Anxiety Inventory [STAI, (93). French version translated by Schweitzer and Paulhan (94)] was used to assess the presence and severity of *anxiety* symptoms. The state anxiety subscale is composed of 20 items rated on a 4-point Likert-like scale from 1 “*not at all*” to 4 “*very much so*” and the trait-anxiety, with also 20 items, from 1 “*almost never*” and 4 “*almost always*.” Higher scores indicate severe anxiety. Cut-offs STAI-S: mild between 36 and 45, median: 46–55, high: 56–65, very high: > 65. Cronbach's alpha for both subscales were 0.91.

*Social anxiety* was assessed using the Liebowitz Social Anxiety Scale self-reported version [LSAS-SR, (95)]. Validated in French by Yao et al. (96), this 24-item scale measures social phobia through two subscales: fear triggered and the avoidance of social situations considering the previous week. Items are rated on 4-point Likert-like scale. A total score can be also calculated by adding the score in each subscale. Higher scores indicated higher levels of social anxiety. Scores: low social anxiety: 56–65, marked: 65–80, severe: 80–95 and very high: > 95. Cronbach's alpha coefficients in this study was 0.88.

The Outcome Questionnaire [OQ®-45.2, (97). French validation by Flynn et al. (98)] was used to evaluate the progress of the course of therapy and the following termination. Composed of 45 items rated on 5-point Likert-like scale, ranging from 0 “*Never*” to 4 “*Almost always*,” this questionnaire contains three subscales: Symptom Distress (SD), evaluating depression and anxiety, Interpersonal Relationships (IR), assessing loneliness, conflict with others and marriage and family difficulties, and Social Role (SR), evaluating the difficulties in the workplace, at school or home duties. A total score can be also calculated. Higher scores suggesting higher functional problems. In the present sample Cronbach's alpha were between 0.77 and 0.95.

*Quality of life* was measured using the World Health Organization Quality of Life [WHOQOL-BREF, (99)]. This 26-item version, rated on a 5-point Likert-like scale, assesses quality of life. Global score and four domains: physical (PHYS), psychological (PSYCH), social (SOC), and environmental (ENVIR) quality of life can be calculated. Higher score means higher perception of quality of life. Cronbach's alpha coefficients in this study were ranged from 0.66 to 0.80 for the subscales and 0.48 for the score global.

Psychological resources were measured using the following instruments presented online with LimeSurvey®.

Participants evaluated their perceived *self-efficacy* with the General Self-Efficacy Scale [GSES, (100). French translation and validation by Dumont et al. (101)]. This 10-item scale assesses the general self-efficacy, optimistic self-beliefs to cope with a variety of difficult demands in life. The items are rated on a 4-point-Likert-like scale going from 1 “*not at all true*” to 4 “*exactly true*.” A higher score indicates a better general self-efficacy. Cronbach's alpha in this sample was 0.94.

*Sense of coherence* was assessed with the 13-item Sense of Coherence Scale [SOC-13, (102). French validation by Gana and Garnier (103)]. Items are rated on a 7-point Likert-like scale, ranging from 1 “*Never have this feeling*” to 7 “*always have this feeling*.” Three components can be distinguished: comprehensibility, manageability and meaningfulness. A high score expresses a strong sense of coherence. Cronbach's alpha in this sample was 0.85.

*Self-compassion* was evaluating using the Self-compassion scale Short Form [SCS-SF, (104). French translation and validation by Kotsou and Leys (105)]. Composed of 12 items, rated on a 5-point Likert-like scale from 1 “almost never” to 5 “almost always,” this scale measures through 6 subscales individual's level of self-kindness, self-judgement, common humanity, isolation, mindfulness, and over-identification. A total score, can be also computed. A total score is calculated by taking the mean of the 12 items after reverse scoring negatively worded items. Higher scores suggesting higher level of self-compassion. Cronbach's alpha in this study was 0.86.

The Multidimensional Scale of Perceived Social Support [MSPSS, (106)] was used to assess *perceived social support*. This 12 items scale evaluated three dimensions: Family, Friends, and Significant others. The items are rated on a 7-point Likert-like scale from 1“*very strongly disagree*” to 7 “*very strongly agree*.” A total score can be calculated, the higher the score the higher the perceived social support. Cronbach's alpha in this study was 0.93.

### Statistical Analysis

#### Determination of Adequate Sample Size

To determine the optimal sample size, we performed an a priori power analysis using G^*^Power [Version 3.1.9.2, (107)] and computed an expected medium effect size based on the meta-analysis of Regehr et al. (60) for an ANOVA with 2 measurement points, 2 groups and between and within factors interaction. We obtained a sample size of *N* = 54. In addition, we estimated a drop-out rate of 15% based on the results of similar intervention program (80), leading to an adequate sample size of 64 participants.

#### Analyses of Intervention Effects

The statistical analyses were computed with IBM® SPSS® Statistics 25 (IBM Corp. Released 2017). Two-way mixed ANOVAs were computed. The within and between independent variables were, respectively, time (pre/T1 vs. post/T2), and group (intervention vs. control), both with two levels. The dependent variables are the different outcome scores of psychological symptoms, quality of life and psychological resources.

We analyzed our data using the per-protocol (PP) approach (108). In that respect, we calculated the ANOVA analyses but only with data from participants who participated in at least five of the eight intervention sessions and who answered the post-treatment measures. Considering the completion of post-treatment measures and according to the dependent variable considered, the sample of post-treatment participants for the PP-analyses varies from 56 to 60. *Post-hoc t*-tests were used to analyze the significant effects related to the a priori hypotheses. Effect sizes (Cohen's *d*) were also calculated, using https://www.psychometrica.de/effektstaerke.html.

To increase the confidence of our results, we performed the same analyses considering an intention-to-treat approach (ITT). In the ITT analyses, all randomized participants who completed the pre-treatment assessment (T1) were taken into account, including non-completing participants and those with missing outcomes. Missing data at post-treatment assessment (T2) were dealt by using the last observation carried forward method (LOCF), which in this case correspond to the pre-treatment measure (T1) (108). A total of 64 participants were taken account for these analyses. The differences between the two analyses are reported in the results' section related to the concerned outcomes.

## Results

Participants present in the sessions varies from 5 (1 person) to all session (11 students), a majority of students (70%) attended 7 or 8 sessions, 25% of students were present at 6 and all of them finished the treatment.

Means and standard deviation (*SD*) of total scores and sub-scores are presented in Table 3 for the outcomes variables evaluating the psychological symptoms, quality of life and psychological resources for the PP- sample.

**Table 3:** Descriptive statistics for outcome variables evaluating psychological symptoms, quality of life, and psychological resources.

**Variable**	**T1**				**T2**				**Statistics**	
	**Intervention group**		**Wait-list control group**		**Intervention group**		**Wait-list control group**			
	**Mean**	***SD***	**Mean**	***SD***	**Mean**	***SD***	**Mean**	***SD***	***t***	***p***
**PSYCHOLOGICAL SYMPTOMS AND QUALITY OF LIFE**										
BDI-II	5.96	4.19	6.22	5.50	4.63	4.37	6.69	5.49		ns
STAI-S	31.19	7.42	32.06	9.12	30.33	10.04	34.00	12.35		ns
STAI-T	38.85	7.56	38.97	10.87	33.85	7.24	37.47	9.63	*t_(25) =_*4.11^b^	< 0.001
LSAS-SR - Total	38.27	19.72	41.44	25.15	31.12	22.05	39.91	24.97		ns
SCL-27-PLUS										
Social phobia	1.30	0.75	1.68	0.93	0.90	0.74	1.48	0.86		ns
Vegetative	1.07	0.55	1.23	0.56	0.76	0.55	1.04	0.50		ns
Pain	1.43	0.71	1.61	0.66	0.88	0.57	1.34	0.69	*t_(58) =_*−2.77^a^	0.008
Agoraphobic	0.45	0.53	0.61	0.51	0.39	0.52	0.48	0.50		ns
Current depression	0.52	0.47	0.62	0.61	0.50	0.75	0.71	0.61		ns
OQ45.2 - Total	41.08	20.60	45.35	23.82	32.65	15.40	42.39	23.71		ns
SD	21.96	11.27	25.55	13.63	16.27	8.19	22.97	14.08		ns
IR	10.00	6.70	9.61	6.80	7.61	5.49	9.97	6.14	*t_(25) =_*2.47^b^	0.021
SR	8.50	4.00	9.30	5.11	8.19	4.27	8.52	4.87		ns
WHOQOL-Bref - Global	17.15	1.97	17.19	2.59	17.62	2.40	17.25	2.68		ns
PHYS	16.56	1.89	16.09	2.52	17.12	1.97	16.54	2.50		ns
PSYCH	14.62	2.24	14.48	2.42	15.46	1.77	14.31	2.32	*t_(55) =_*2.05^a^	0.045
SOCIAL	15.36	2.83	16.17	2.93	16.33	2.73	15.74	3.41		ns
ENVIR	16.83	2.03	16.34	2.32	17.37	1.20	16.74	2.29		ns
**PSYCHOLOGICAL RESOURCES**										
SOC - Total	65.89	9.95	66.28	12.44	71.04	8.04	67.09	11.70	*t_(25) =_*−0.48^b^	0.002
SCS-SF - Total	3.09	0.51	3.09	0.85	3.49	0.59	3.16	0.98	*t_(24) =_*−0.61^b^	0.001
GSES	31.40	6.05	31.78	7.35	34.72	3.37	33.25	5.05		ns
MSPSS	6.05	0.83	6.04	0.94	6.21	0.68	5.82	1.31		ns

Mean (SD) value at pre- (T1) and post-treatment (T2) by treatment condition (intervention group vs. wait-list control group) in the PP-sample (n = between 56 and 60)

SD, Standard Deviation; BDI-II, Beck Depression Inventory-II; STAI-S and STAI-T, Spielberger State-Trait Anxiety Inventory; LSAS-SR, Liebowitz Social Anxiety Scale; SCL-27-plus, Symptom Checklist; OQ45.2, The Outcome Questionnaire 45.2; SD, Symptom Distress; IR, Interpersonal Relationships; SR, Social Role; WHOQOL-Bref, World Health Organization Quality of Life-Bref; PHYS, Physical; PSYCH, Psychological; SOC, Social; ENVIR, Environmental; SOC, Sense of Coherence Scale; SCS-SF, Self-compassion Scale Short Form; GSES, General Self-Efficacy Scale; MSPSS, Muldimensional Scale of Perceived Social Support.

a Post-hoc independent t-test

b Post-hoc paired t-test

^*^p < 0.05.

### Pre-post Treatment Analyses

#### Psychological Symptoms and Quality of Life

**Figure 2:**
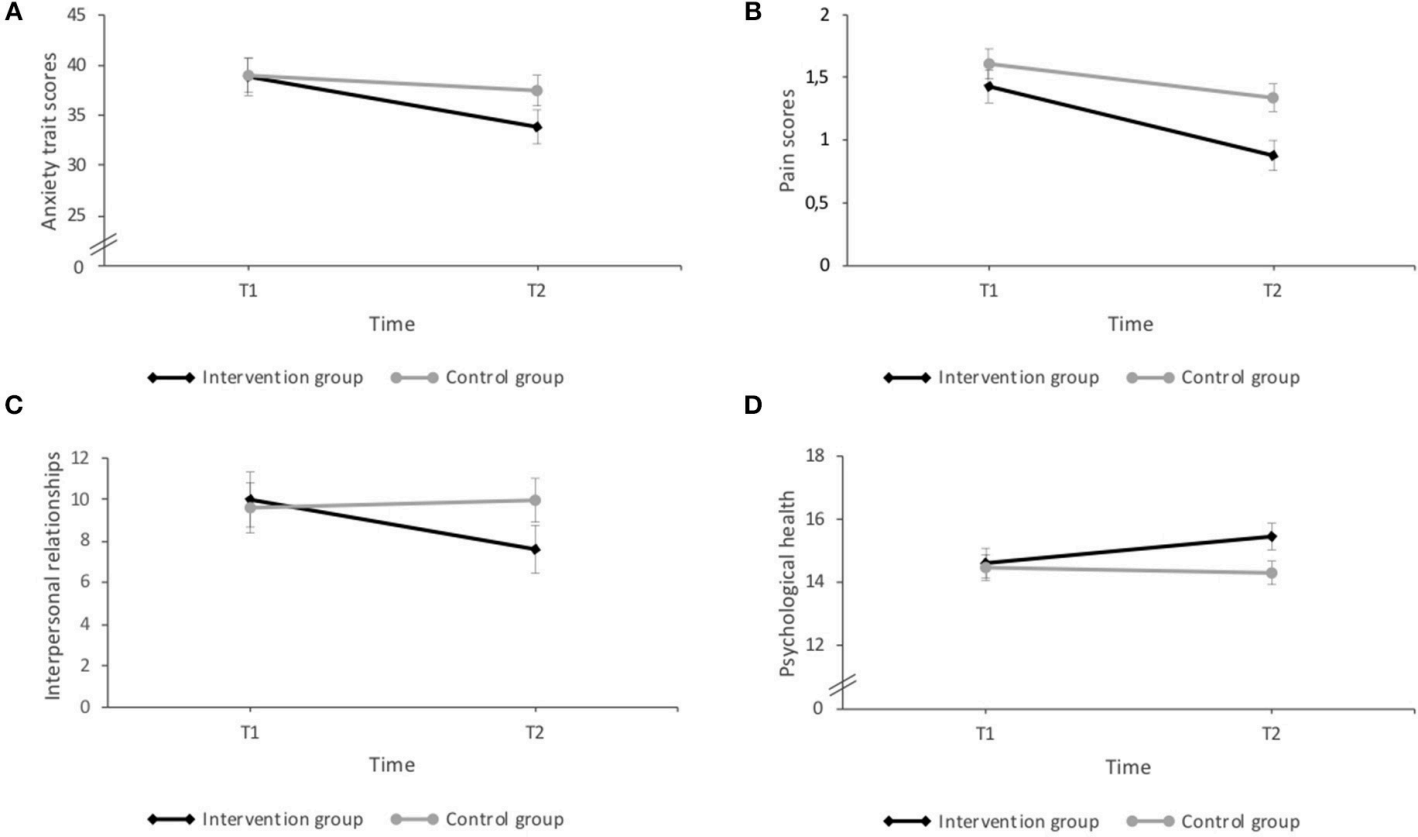
Results of the interaction effects (time x intervention) in psychological symptoms and quality of life: **(A)** Anxiety trait: the intervention group reported significantly less anxiety trait at T2 compared with T1. **(B)** Pain symptoms: the intervention group reported significantly lower pain symptoms at T2 in comparison to the wait-list control group; both groups showed also significantly less pain symptoms at T2 than at T1. **(C)** Interpersonal relationships: the intervention group reported significantly lower interpersonal difficulties at T2 than at T1. **(D)** Psychological quality of life: the intervention group reported significantly higher scores in the psychological quality of life perceived at T2, compared to the wait-list control group. The intervention group showed also significantly higher scores at T2 than at T1. Errors bars represent standard errors. *P* < 0.05.

Results of the mixed ANOVA's for the psychological symptoms and the quality of life (Figure 2), showed a significant interaction effect between time and intervention in the trait anxiety levels measured with the STAI [*F*_(1, 56)_ = 4.87, *p* = 0.031, η^2^ = 0.08]. *Post-hoc* paired *t*-test revealed that students who participated in the intervention group reported significantly less anxiety traits at T2 in comparison to T1(*p* < 0.001; *d* = −0.68). No other significant effects were found (all *p* > 0.05).

The ANOVA analyses for the SCL-27-plus showed a significant interaction effect (time x intervention) for the pain dimension [*F*_(1, 58)_ = 4.80, *p* = 0.033, η^2^ = 0.08] evaluated by *post-hoc* independent *t*-tests indicated that the mean score on the pain perception in the intervention group was significantly lower at T2 in comparison to the wait-list control group (*p* = 0.008; *d* = 0.73). Furthermore, paired *post-hoc t*-test, revealed that the students who participated in the intervention revealed significantly less pain symptoms at T2 than at T1 (*p* < 0.001; *d* = −0.85), but also the wait-list control (*p* = 0.002; *d* = −0.40). For the other dimensions, significant effects of time were found for the agoraphobic [*F*_(1, 58)_ = 4.27, *p* = 0.043, η^2^ = 0.69], and vegetative symptoms [*F*_(1, 57)_ = 16.33, *p* < 0.001, η^2^ = 0.22]. These results indicated that the agoraphobic and the vegetative symptoms scores were significantly lower in both groups at T2 that at T1. Larger significant time main effect was found for the dimension of social phobia [*F*_(1, 57)_ = 17.33, *p* < 0.001, η^2^ = 0.23], and moderate for group main effect [*F*_(1, 57)_ = 5.56, *p* = 0.022, η^2^ = 0.09], indicating that the scores at T2 were significantly lower that at T1, and that the intervention group had significantly lower scores than the wait-list control group in this dimension.

The ANOVA analyses of the outcome questionnaire (OQ-45.2) showed a significant interaction effect between time and intervention for the IR sub-scores [*F*_(1, 55)_ = 4.71, *p* = 0.034, η^2^ = 0.08]. *Post-hoc* paired *t-*tests revealed that participants of the intervention group report significant lower difficulties in interpersonal social relationships at T2 than at T1 (*p* = 0.021; *d* = −0.39). A significant main effect of time was found for the sub-score of SD [*F*_(1, 55)_ = 13.05, *p* = 0.001, η^2^ = 0.19] and for the total score [*F*_(1, 55)_ = 8.17, *p* = 0.006, η^2^ = 0.13]. No other significant effects were found.

With regard to the quality of life (WHOQOL-BREF), the results of the ANOVA's indicated a significant interaction effect between time and intervention for the dimensions psychological [*F*_(1, 55)_ = 4.65, *p* = 0.035, η^2^ = 0.08] and social of the quality of life [*F*_(1, 55)_ = 4.81, *p* = 0.033, η^2^ = 0.08]. *Post-hoc* independent *t*-tests revealed that the mean score in the dimension psychological quality of life was significantly higher in the intervention group at T2, compared to the wait-list control group (*p* = 0.045; *d* = −0.56), and paired *t-*test showed also that the intervention group revealed significantly higher scores at T2 compared with T1 (*p* = 0.032; *d* = 0.42). No simple effects were found for social health quality (*p* >0.05). A significant moderate main effect was found for time in the physical dimension [*F*_(1, 55)_ = 5.55, *p* = 0.022, η^2^ = 0.09], the scores in this dimension were significant higher at T2 in comparison with the score at T1 for both groups. No other significant effects were found in the analysis of the other dimensions (physical and environment) and in the global score of quality of life (all *p* > 0.05).

The ANOVA analyses for the BDI-II and the LSAS-SR showed no significant interaction effects between time and intervention for the scores of depression [*F*_(1, 57)_ = 1.91, *p* = 0.173] or social anxiety. A significant main effect was found for time [*F*_(1, 56)_ = 6.74, *p* = 0.012, η^2^ = 0.10], but not for group [*F*_(1, 56)_ = 1.02, *p* = 0.317] for social anxiety. No other significant results were found.

Results analyses of the psychological symptoms and quality of life outcome variables using the ITT-sample were similar as the findings in the PP-sample only for the interaction effects between time and intervention for the Interpersonal relationship (IR) sub-score of the outcome questionnaire (OQ-45.2) [*F*_(1, 62)_ = 4.08, *p* = 0.048, η^2^ = 0.06], and for the dimensions psychological [*F*_(1, 60)_ = 4.08, *p* = 0.048, η^2^ = 0.06] and social [*F*_(1, 60)_ = 4.48, *p* = 0.038, η^2^ = 0.07] quality of life (WHOQOL-BREF). Similar to the analyses in the PP-sample no significant effects were found in the ANOVA analyses of the BDI-II, and the LSAS-SR in the ITT-sample. However, contrary to the analyses in the PP-sample, the analyses of the ITT-sample for the trait anxiety (STAI) and for the pain dimension of the SCL-27-plus, showed no significant interaction effects between time and intervention (for details see **Supplementary Material**).

#### Psychological Resources

**Figure 3:**
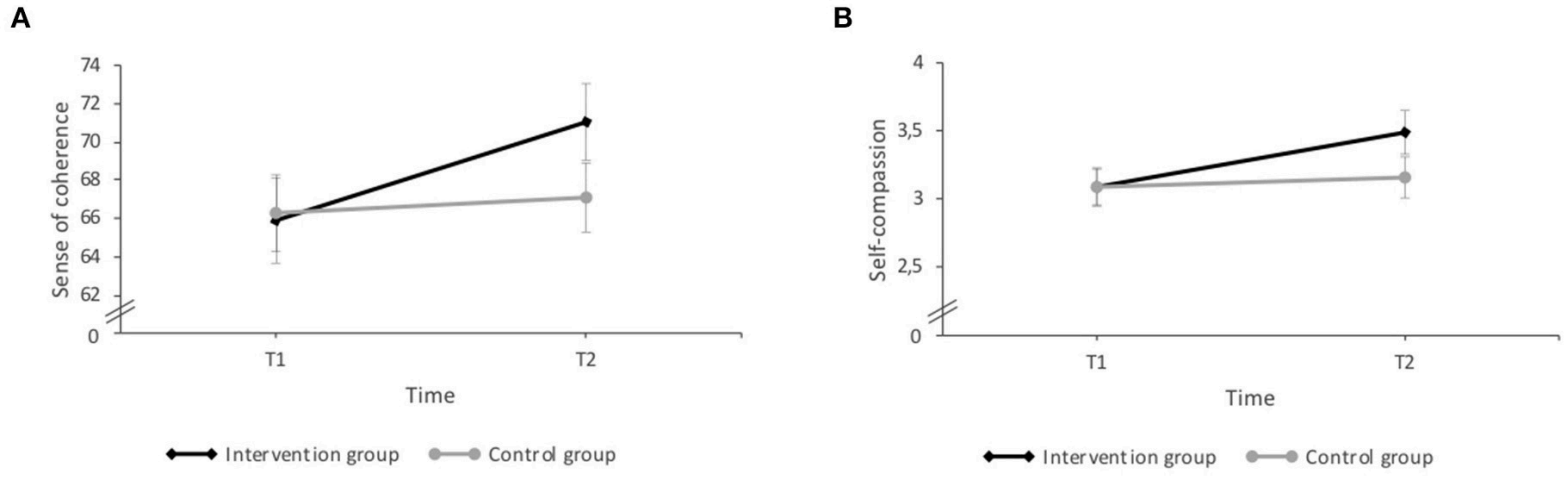
Results of the interaction effects (time × intervention) in psychological resources: **(A)** Sense of coherence: the intervention group reported significantly higher levels of sense of coherence at T2 than at T1. **(B)** Self-compassion: the intervention group revealed significantly higher scores in self-compassion at T2 compared with T1. Errors bars represent standard errors. *P* < 0.05.

The ANOVA analyses of the SOC-13 and SCS-SF, yielded a significant interaction effect between time and intervention for sense of coherence [*F*_(1, 56)_ = 5.50, *p* = 0.023, η^2^ = 0.09] and self-compassion [*F*_(1, 54)_ = 4.64, *p* = 0.036, η^2^ = 0.08]. *Post-hoc* paired *t*-tests indicating that the participants of the intervention group showed significant higher levels of sense of coherence and of self-compassion at T2 than at T1 (*p* = 0.002; *d* = 0.57; *p* = 0.001; *d* = 0.72, respectively) (Figure 3).

With regard to self-efficacy, the ANOVA analyses of the GSES revealed no significant interaction effect between time and intervention [*F*_(1, 55)_ = 1.35, *p* = 0.251] but a large significant main effect for time [*F*_(1, 55)_ = 9.01, *p* = 0.004, η^2^ = 0.14] but no for group. The score of self-efficacy was significant higher after the treatment for both groups, in comparisons with the scores at T1. ANOVA analyses of the MSPSS showed no significant interaction effects or main effects for time and group for the perceived social support (all *p* > 0.05).

Considering the ANOVA analysis of the psychological resources taken into account in the ITT-sample, the results are similar to the PP-sample results (for details see the **Supplementary Material**).

## Discussion

This study aimed at evaluating the short-term effects of a multidimensional stress prevention program on psychological symptoms, well-being, and psychological resources in university students. The most remarkable results are the improvement in quality of life, and psychological resources, including sense of coherence and self-compassion, as well as the decrease of specific psychological symptoms, such as anxiety, pain, and interpersonal problems, in the intervention group compared with the wait-list control group.

With regard to the psychological symptoms, we found, as expected, significant decrease in anxiety scores in the intervention group as compared to the wait-list control group. These findings are consistent with previous research analyzing stress prevention programs among university students (60, 109, 110). A meta-analysis about the evaluation of stress reduction interventions among medical university students, indicated that mindfulness-based stress reduction, meditation techniques and self-hypnosis are effective in reducing anxiety (111). Many studies have reflected the improvement of anxiety (60). Surprisingly, our findings reflected a reduction in trait-anxiety but not in state-anxiety. This could be explained by the composition of our sample, we included only participants without psychopathological complains, which could have affected their level of state anxiety. Furthermore, the post-intervention measurements were always at the end of the semester, during the review period, and they were closed to the exams. This could also have an effect on the levels of anxiety because it is known as an anxious and stressful period for students.

The significant reduction in pain symptoms in the intervention group is in line with our hypothesis of a diminution of psychopathological symptoms. This is particularly important as university students were shown to report increased psychosomatic symptoms (30). This is also interesting as other validated intervention programs in students did not find any effect on somatic and/or psychosomatic symptoms. We should also note that the control group reported a reduction in the pain score, but to a lesser extent. The lack of significant results for the other dimensions of the SCL-27-plus could be explained again by the fact that we included only asymptomatic participants, i.e., without clinically significant psychopathological symptoms. Therefore, it is not surprising that we did not find any significant changes on measures of psychopathological symptoms. This could also explain the lack of differences observed for the depression scores.

The intervention improves, as expected, the functional level of the participants, but only in the domain concerning the interpersonal problems in the intervention group, compared to the wait-list control group. These results are very relevant as recent researches indicated that psychosocial factors, such as perceived social support, and resilience, are protective factors of mental health in university students (21). Stress levels are related also with social isolation [e.g., among law students (112, 113)], and also with not having a satisfying relationship with the family and friends in university students (114). Interpersonal stress was also associated with depression, anxiety and somatization (115), and suicide risk (116, 117) in university students. To better adjust at the university context campus connectedness (118), family and peer support, and satisfying relationships are important (38, 119) for university students. These psychosocial factors are a mediator between stress and health consequences (120). Therefore, as predicted, we found an improvement in the quality of psychological health. These findings are consistent with research indicating the link between stress and quality of life in university students, and the importance to reinforce the mediators between them, such as personal and psychological resources (54). Furthermore, in recent studies, the use of individual strength in university students showed benefits in mental health among students (121) and was positive related with positive affect, self-esteem, and vitality, and negatively with stress and negative affect (122).

Very interestingly, the short-term effects of our intervention indicate significant increases in specific psychological resources, including higher levels of sense of coherence and self-compassion after the intervention in the intervention group than the wait-list control group. These results are consistent with other findings suggesting that sense of coherence is an indicator of resilience and can be regarded as an attitude or predisposition promoting health and resilience by using different personal resources (123, 124). Higher sense of coherence was found to be associated with less stress and better quality of life in students (52, 54, 123, 125). In addition, improvement in the sense of coherence is interesting because this concept is comprised as an attitude or predisposition (126). In this sense, it is important because it will allow the participants to change their attitude toward future stressful events and other situations (124). Previous research indicates the relationship between self-compassion and psychological well-being in university students (127, 128). In view of previous research that showed the importance of personal resources (54), like optimism (129), self-efficacy, and resilient coping (3) related with decreased perceived stress, these results are important for an intervention aiming at reducing stress and increasing resources in a students' population. Nevertheless, further long-term studies have to be done to investigate the potential protective effect of these increased personal resources against stress.

Contrary to our expectations and previous studies, social support and self-efficacy are not improved after the participation in the program (130, 131). Self-efficacy is one the most important predictors of distress (25), but also an important personal resource to reduce the effects of stress in well-being (132), like social support (133, 134).

Taken together these results indicated promising short-term effects of our program. Specifically, because it increases some important resources against stress but also because the participation in the program have effects in psychological symptoms in an asymptomatic sample. The results are consistent with the objective of this multidimensional intervention, which is not only to focus on stress reduction, but also to improve some personal skills and psychological resources to prevent future stressful situations. The replication of the results across different samples, per-protocol and intention-to-treat, suggest that our program have an important short-effect on psychological symptoms and quality of life, particularly interpersonal relationship difficulties, psychological, and social quality of life, but also in personal resources (sense of coherence and self-compassion). Unfortunately, we cannot specify exactly which dimension of our program has a particular effect on which variable, but our results show that the entire program has an effect on psychological symptoms, well-being and psycho-social resources.

Some limitations deserve to be taken into consideration. First, the sample was composed by a majority of students of the University of Fribourg, females and studying psychology, which limits the generalization of our results. The gender disparity does not allow to compare the differences in the short-term effects of our program between men and females. Bachelor students in psychology had an additional motivation factor for their participation, they could receive experimental points instead of financial reimbursement in order to meet the requirements of the bachelor studies. A second limitation is the control group chosen, the wait-control list. A better control group would be an active one, in this sense it could be interesting to evaluate the effects of our program with an already well-validated stress intervention (i.e., cognitive, behavioral, or mindfulness-based), in order to distinguish more in detail, the effect of the multidimensionality. Third, the use of self-report instruments can lead to memory bias and greater subjectivity in the responses, specially the length of our online questionnaires (~1 h) may have led to less accurate answers due to fatigue, even if participants could take breaks. Fourth, the relatively small sample size can be also a factor to take into account in the limitations. Fifth, we did not control for past finished psychological or drug treatments. However, we controlled that none of the participants was receiving a treatment (neither drug or psychological) at the time of the study. A last limitation is that we cannot completely rule out that a person external to the study has filled the online questionnaires using the personal codes.

However, our study also has some specific strengths, including for instance the use of a randomized controlled design. There is also very little drop-out related to the intervention. It seems that the participants who engaged in the program also stayed until the end since all participants have completed the program; and 70% of them participated to all sessions. Future studies have to analyze the medium and long-term effects of our program in a larger healthy sample, but also, evaluate the effects in clinical samples. Furthermore, it could be interesting to evaluate the effects of this program using biomarkers or daily life assessments.

In conclusion, our findings provide very promising preliminary evidence of the efficacy of our multidimensional stress prevention program, not only in the reduction of psychological symptoms, but also in the improvement of well-being and some important psychological resources increasing the resilience to stress. In that way, we can also define our program as a resource-activating intervention.

## Author Contributions

RR, CG, MG, and CM-S contributed to the conception and design of the study and were involved in the interpretation of the data. RR, CG, and MG contributed to the conception of the measurements and collection of data. RR performed the statistical analyses. RR and CM-S wrote sections of the manuscript. All authors contributed to manuscript revision, read, and approved the submitted version.

### Conflict of Interest Statement

The authors declare that the research was conducted in the absence of any commercial or financial relationships that could be construed as a potential conflict of interest.
